# Tailoring Artificial Mode to Enable Cofired Integration of Shear‐type Piezoelectric Devices

**DOI:** 10.1002/advs.202001368

**Published:** 2020-07-06

**Authors:** Jikun Yang, Qiang Huan, Yang Yu, Jingen Wu, Zhaoqiang Chu, MohammadJavad PourhosseiniAsl, Faxin Li, Shuxiang Dong

**Affiliations:** ^1^ Department of Materials Science and Engineering College of Engineering Peking University Beijing 100871 P. R. China; ^2^ Beijing Key Laboratory for Magnetoelectric Materials and Devices (BKL‐MEMD) Peking University Beijing 100871 P. R. China; ^3^ LTCS and Department of Mechanics and Engineering Science College of Engineering Peking University Beijing 100871 P. R. China

**Keywords:** actuation, cofired manufacture, integrated piezoelectric devices, sensing, shear mode, structural health monitoring

## Abstract

Low‐temperature cofired ceramic technology is the prerequisite for producing advanced integrated piezoelectric devices that enable modern micro‐electromechanical systems because of merits such as high level of compactness and ultralow drive voltage. However, piezoceramic structure with shear‐type outputs, as a most fundamental functional electronic element, has never been successfully fabricated into multilayer form by the cofired method for decades. Technical manufacture requirements of parallel applied electric fields and polarization are theoretically incompatible with intrinsically orthogonal orientations in naturally occurring shear modes. Herein, inspired by the philosophy of building metamaterial from identical unit cells, an artificial prototype device with distinctive patterned electrodes and arrayed piezoceramic subunits is designed and fabricated, which is proved to perfectly generate synthetic face shear deformation. At the same drive voltage, an enhanced shear‐type displacement output by over an order of magnitude is observed beyond previous d_15_‐mode bulk elements. Further results of guided wave‐based structural health monitoring and force sensing confirm that the methodology wipes out a tough piezoelectric technique barrier, and promises to fundamentally enlighten advances of integrated shear‐mode piezoelectric devices for augmented actuation, sensing, and transduction applications.

## Graphical abstract

This work reports an ingenious metamaterial‐inspired methodology to first realize cofired manufacture of multilayer shear‐type piezoelectric devices, which is a long‐term challenge in piezoelectric techniques. The artificially prototype d_36_‐mode device shows enhanced shear‐type displacement output by over 20 times beyond previous d_15_‐mode bulk one at the same drive voltage, and leads to obviously augmented performance for actuation, sensing, and transduction applications.

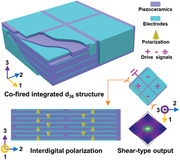

## 1 Introduction

Piezoelectric actuators or micromotors driven by single crystals or piezoceramics enable precise positioning and actuation at meso or nanoscale level, which shines a lot at advanced electronic industries such as minimally invasive surgical apparatus,^[^ ^1^ ^]^ semiconductor photolithography positioners,^[^ ^2^ ^]^ laser or camera modules,^[^ ^3^ ^]^ and other micro‐electromechanical systems (MEMS).^[^ ^4^, ^5^, ^6^, ^7^ ^]^ The intrinsic electromechanical coupling effect of piezoelectric materials originates from their lattice level, so piezoelectric actuators as well as sensors, especially integrated type based on multilayer structures, possess the merits of fast response, antielectromagnetic interference, high power density, and tiny dimensions.^[^ ^8^, ^9^, ^10^, ^11^, ^12^, ^13^, ^14^ ^]^ As a fundamental working mechanism, shear mode of piezoelectric elements plays indispensable roles in nanopositioning workbench,^[^ ^5^, ^15^, ^16^ ^]^ distributed intelligent sensing,^[^ ^17^, ^18^, ^19^ ^]^ energy conversion apparatus,^[^ ^20^, ^21^ ^]^ and other electromechanical devices.^[^ ^14^, ^22^, ^23^ ^]^ However, conventional excitation methods of shear mode always rely on expensive and brittle piezoelectric single crystals^[^ ^15^, ^16^, ^17^, ^18^, ^19^ ^]^ (d_36_ mode) or bulk ceramics^[^ ^14^, ^22^, ^24^, ^25^ ^]^ (mainly d_15_ mode), which all inevitably require high operate voltages to offer substantial displacement outputs and lead to poor service robustness from mechanical fragility or easy domain switching problems. To realize shear‐type piezoelectric elements with efficient outputs compatible with modern integrated microelectronics,^[^ ^5^, ^6^, ^14^ ^]^ these tough obstacles are urgently expected to be overcome.

Generally, piezoelectric devices are designed into polymorphic configurations such as unimorph,^[^ ^26^, ^27^ ^]^ bimorph,^[^ ^28^, ^29^ ^]^ moonie, or other metal/ceramics laminated forms^[^ ^30^, ^31^ ^]^ to meet specific actuation and sensing demands.^[^ ^32^, ^33^ ^]^ The monolith multilayer structure is able to accumulate piezoelectric strain or charge of each superimposed piezoceramic layer in a very limited volume.^[^ ^34^, ^35^, ^36^ ^]^ Stacked‐disk method^[^ ^35^ ^]^ and piezoceramic cofired method^[^ ^36^ ^]^ are main approaches to obtain multilayer devices. Superior to the former one, cofired and tape‐casting technologies can realize ultrathin single active layer (up to micros), much higher level of mechanical integration and ultralow working voltage (only tens of volts or lower).^[^ ^37^, ^38^ ^]^ Without adhesives restriction, structures also present splendid mechanical strength. The cofired manufacture technique is also compatible to those of mature multilayer ceramic capacitors (MLCC), and already become the premise of industrial mass production of piezoelectric microelectronic elements at low cost.^[^ ^36^, ^37^, ^38^, ^39^ ^]^ If shear‐type piezoelectric devices can be fabricated into compact multilayer structures based on cofired method, these tough service deficiencies promise to be broken through. Nevertheless, as a main challenge since this influential technology invented decades ago, cofired shear‐type piezoceramic structures have never been designed nor realized yet.

The basic materials used for cofired devices are piezoceramics.^[^ ^5^, ^37^ ^]^ Their intrinsically high transversely isotropic symmetry leads to only three independent nonzero piezoelectric strain coefficients, namely, d_31_ (d_32_), d_33_, and d_15_ (d_24_). As the sole shear mode, applied electric field and initial poling direction of d_15_ mode are in orthogonal orientations, and intermediate electrodes need polishing and re‐preparing after polarization.^[^ ^40^, ^41^ ^]^ Whereas for cofired multilayer structures, the electrodes should be cosintered with piezolayers in one procedure and will not support reconstruction.^[^ ^37^, ^39^ ^]^ Single crystals of lower intrinsic symmetry may excite face shear (or d_36_) mode, where the polarization is parallel to electric fields.^[^ ^15^, ^16^ ^]^ However, crystals essentially do not allow to be prepared as functional slurry for tape‐casting process and cofired treatment. In our recent work, we revealed that apparently macroscopic broken symmetry of piezoceramics may be artificially established and further developed a methodology to design electromechanical metamaterials with overall nonzero effective coefficients beyond nature.^[^ ^40^ ^]^ Inspired by the prediction that apparent face‐shear mode of normal ceramics is supposed to be excited by arrayed functional subunits with programmed anisotropic polarization and external electric fields, we originally design and fabricate an artificial multilayer structure, and cofired integrated shear‐type devices is expected to be realized for the first time.

**Figure 1:**
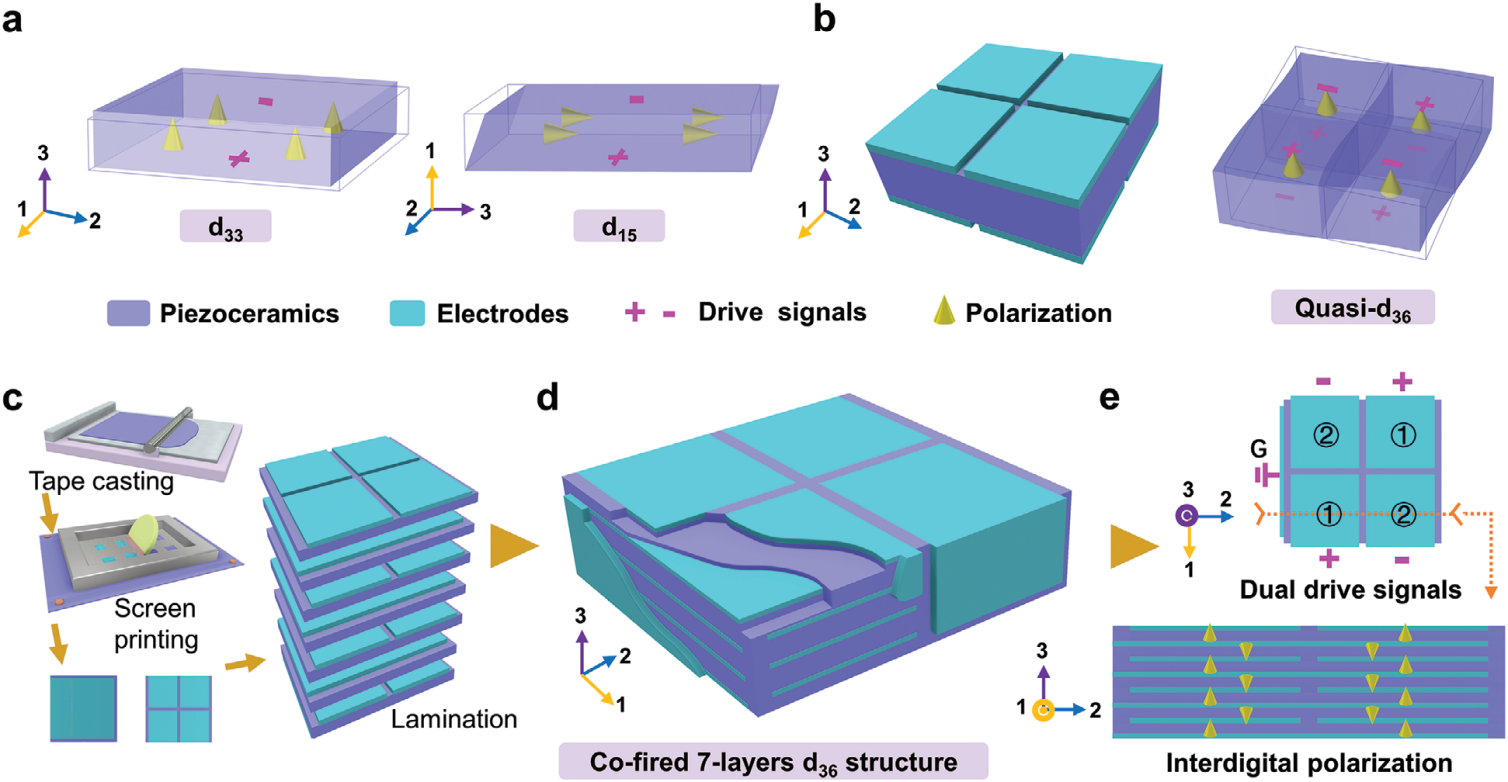
Schematic design and fabrication processes of cofired shear‐mode piezoelectric structure. a) Naturally occurring d_33_ normal strain mode features parallel polarization and electric field, while in‐plane d_15_ shear mode has perpendicular orientations. Similar orthotropic relationship in thickness poled d_15_ and in‐plane poled d_24_ shear mode is shown in Figure S1 (Supporting Information). b) Artificial structure composed of four d_31_ normal strain subunits will synergistically lead to quasi‐d_36_ face‐shear deformation. Based on improved classical fabrication procedures, c) novel structure composed of patterned electrodes and arrayed piezoceramic subunits is designed, and a d) cofired 7‐layer prototypical structure is created. e) With interdigital polarization and under reverse phase dual drive signals, the cofired multilayer structure will exhibit d_36_ face shear deformation.

In this study, an unprecedented 7‐layer piezoelectric structure based on tape casting and cofired technologies is designed and fabricated. Its distinctive interdigital electrodes are partitioned to form patterned morphologies (**Figure** **1**). Under anisotropic dual drive signals, arrayed functional subunits of the cofired device will synergistically excite equivalent quasi‐d_36_ face shear deformation. A measured huge transverse displacement output enhanced by over 20 times than traditional d_15_ mode is obtained at the same drive voltage. Its practical performances for structure health monitoring and as force detection transducers are experimentally investigated, and the results demonstrate that the multilayer structure can sharply decrease the operating voltage of piezoelectric actuators, but also amplify the charge response of piezoelectric sensors. Considering other crucial advances such as ultrahigh integration level and perfectly industrialization compatibility, the novel cofired multilayer structure is expected to greatly update conventional shear‐type piezoelectric devices.

## 2 Results and Discussion

### 2.1 Design and Fabrication of Cofired Shear‐mode Piezoelectric Structure

Traditional cofired multilayer piezoceramic devices with normal strain work on longitudinal extension (or d_33_) mode (Figure 1a). In essence, cofired structure compulsively requires both polarization and applied electric fields along with the thickness direction. Unfortunately, the only naturally occurring side‐plane shear (in‐plane or thickness poled d_15_) modes or in‐plane face‐shear (or d_24_) mode possesses perpendicular polarization and electric field (Figure 1a and Figure S1, Supporting Information). To enable shear‐type multilayer structures, an artificial face‐shear (or d_36_) mode of single‐layer piezoceramics is initially developed. As illustrated in Figure 1b, a square piezoelectric plate polarized along with thickness direction is divided into four smaller arrayed subunits by divisional electrodes. When applied diagonally inverse‐phase drive signals, every pair of subunits should work on d_31_ extensional or contractional modes, respectively.^[^ ^21^, ^40^, ^41^ ^]^ Their combined action will lead to an equivalent in‐plane shear deformation, which is quasi‐d_36_ face‐shear mode (see demonstration in Section S1 in the Supporting Information).

Similar to manipulating alignments and synergistic effect of meta‐atoms in metamaterial,^[^ ^42^, ^43^ ^]^ the key design of multilayer d_36_ shear‐mode structure is the arrangement way and resultant action of its ordered subunits. Figure 1c,d illustrate the fabrication processes, patterned layers and lead approaches in detail. A kind of raw PZT‐5H piezoceramic powder (Figure S2, Supporting Information) is prepared as uniform slurry with functional additives by ball‐mixing (see the Experimental Section), and then primary thick films are formed by tape casting. To construct multilayer structures, interdigital electrodes are designed as two types of patterned morphologies and prepared by screen printing. Besides normal fully covered electrodes, other half ones are partitioned into four square subunits. Ordered lamination and hot‐pressing treatment of arrayed subunit sheets will get a monolithic green body. After cofired procedure for crystallization, a unibody 7 layers shear‐mode structure is finally obtained (Figure 1d). Figure 1e schematically shows the interdigital polarization and drive methods. With fully covered electrodes behaving as ground port and partitioned ones driven by diagonally dual inverse‐phase signals, these arrayed subunits will synergistically exhibit quasi‐d_36_ face shear deformation. Remarkably, polarization and external voltages of the novel structure are totally in parallel directions, making it completely compatible with industrialized cofired technologies.

**Figure 2:**
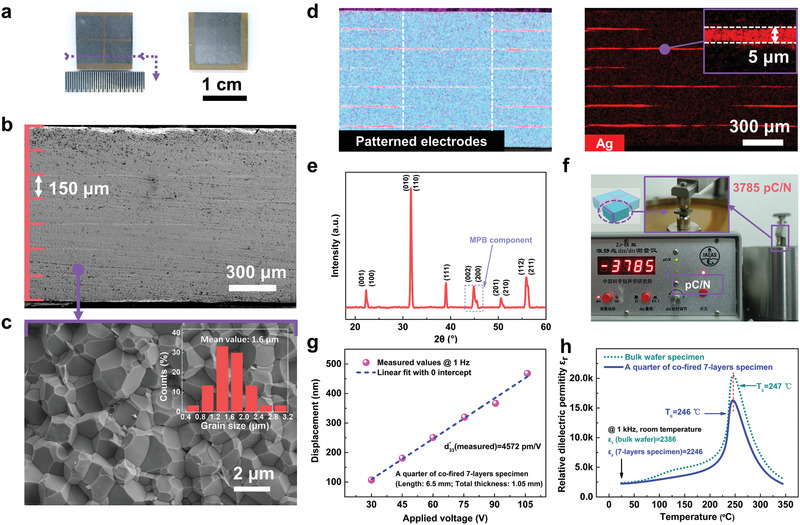
Morphology and inner structure verification, piezoelectric response, and dielectric properties of the prototype cofired 7‐layer device. a) Picture of the front and back main surfaces of the fabricated specimen. b) SEM images of macroscopic or microregion cross‐section reveal integrated 7 uniform piezoceramic layers with thickness around 150 µm, and c) dense structure of fine grain crystallization. d) EDS results show perfectly embedded thin interdigital (ID) electrodes (5 µm), flat interfaces and ideal element distribution as designed. e) XRD result reveals a pure phase of polycrystalline perovskite and coexistence of tetragonal phase and rhombohedral phase, which is the mark of MPB component. f–h) A quarter of the cofired 7‐layer specimen is cut off and investigated to guarantee fine piezoelectric nature. f) Small‐signal piezoelectric coefficient d_33_ is tested to be 3785 pC N^−1^, and g) the relationship between displacement outputs and applied voltages is basically linear with effective large signal $d_{33}^{*}$ calculated as high as 4572 pm V^−1^. h) Temperature dependence variation tendency of measured relative dielectric permittivity of the quarter and a single‐layer wafer is consistent, which ensures the thermal stability of cofired specimen.

The appearance and geometrical dimensions of the obtained prototypical specimen are illustrated in **Figure** 2a and Figure S3 in the Supporting Information. To verify the fabrication reliability, its morphologies, phase structure and other basic electromechanical and dielectric properties are characterized and expounded. Scanning electron microscope (SEM) and X‐ray diffraction (XRD) results indicate the ceramic layers are perfectly crystalized without obvious pores or cracks, of which the piezoceramic layer thickness and the mean grain size are around 150 and 1.6 µm, respectively (Figure 2b,c,e). Using energy dispersive spectroscopy (EDS) method, the element distribution on the cross section is detected and exhibited in Figure 2d. It is clearly proved that thin straight inner electrodes (with thickness around 5 µm) are well embedded in the main ceramic body with ideal patterned configurations and flat interfaces as initially designed. The partitioned electrodes are well aligned to each other, which is the good precondition for objective synergistic shear deformation. There exists no obvious element diffusion between the two ingredients, which ensures the dielectric and piezoelectric performance.

From the view of metamaterial design principles, the novel integrated structure is composed of stacked subunits of d_36_‐mode single layers. Likewise, it can also be regarded as four elaborate arrays of traditional multilayer d_33_‐mode subunits. To investigate the basic piezoelectric nature of the cofired sample, a quarter part is cut off and characterized (Figure 2f and Figure S4, Supporting Information). Apparently small signal piezoelectric coefficients d_33_ is tested by quasi‐static method to be around 3785 pC N^−1^, which indicates that every active piezoelectric layer is pretty well‐polarized and all successfully electrically connected out in parallel. Unipolar strain response of the quarter part under electric fields ranging from 2 to 7 kV cm^−1^ is shown to be basically linear to applied electric field. From the correlation between displacement outputs and applied voltages, the large signal coefficients $d_{33}^{*}$ is calculated as 4572 pm V^−1^ and consistent with results of small signal one (Figure 2g). Temperature dependence of dielectric behaviors of the quarter part and normal bulk wafer specimen ranging from 25 to 350 °C is measured in Figure 2h. The results indicate the Curie temperature and dielectric variation tendency of cofired sample is almost unchanged compared with bulk one, so robust outputs in wide temperature range are physically guaranteed. By programming distinctive patterned electrodes and ordered piezoceramic subunits, artificial quasi‐d_36_ deformation is developed as basic mechanism, and a prototypical shear‐mode 7‐layer device is originally designed and created by advanced tape casting and cofired technologies.

### 2.2 Modal Analysis and Output Characterization

For naturally occurring piezoceramic elements, there exists a significant inadequacy that limits their shear‐type actuation applications. Both d_15_ or d_24_ mode inevitably requests fairly high driving voltage to offer substantial displacement outputs, and more challengingly, because of the perpendicular polarization orientations, relatively bigger electric fields should easily cause domain switching problem and invalidate practical devices. The novel cofired piezoelectric component features multilayer constitution and parallel polarization, hence its actuation capability is expected to be greatly enhanced and fundamentally solve the deficiency in traditional shear‐type devices. To examine the actual performance, its working modal states and effective outputs are investigated and compared with traditional shear mode by numerical simulation and experimental measurements (geometrical models, computational details and theoretical analyses are explicated in Section S3 of the Supporting Information).

**Figure 3:**
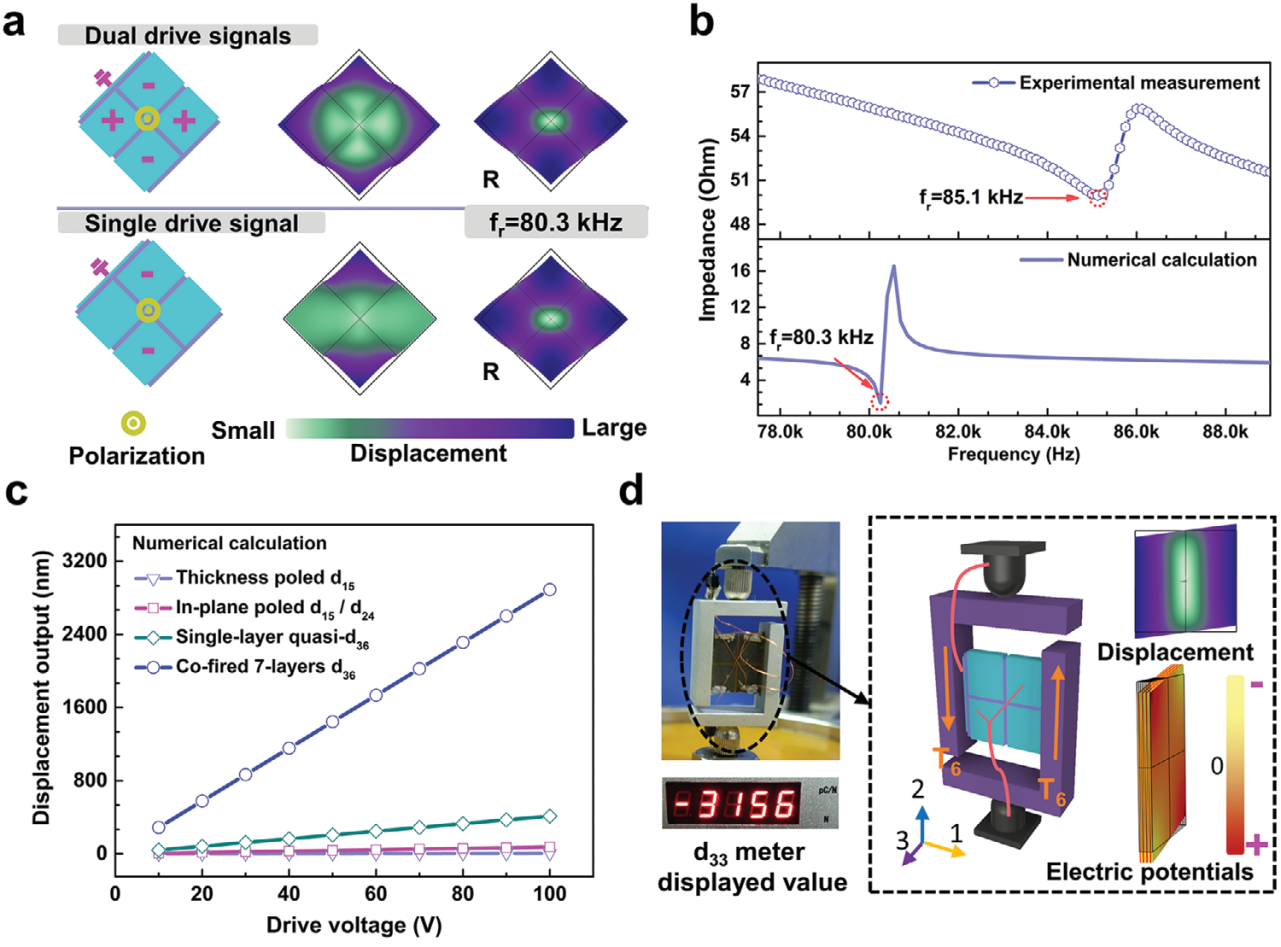
Analyses of deformation behavior, displacement and charge outputs of cofired shear‐mode 7‐layer structure. a) Under both dual or single drive signals, the structure can excite shear‐mode deformation at steady or resonant (with label “R”) frequencies. Response of other typical frequencies is shown in Figure S5 of the Supporting Information. b) Impedance spectrum denotes that measured resonant frequency of the specimen driven by single drive signal matches simulated one well. c) With the same modal dimensions, displacement of conventional naturally occurring shear modes and novel cofired 7‐layer artificial d_36_ mode as a function of drive voltage are calculated. Compared with conventional mode, enhanced values over an order of magnitude are easily obtained. d) Charge response of cofired 7‐layer specimen is detected.

Deformation states of the cofired structure and frequency dependence under two various working ways, namely, single or dual drive signals are simulated by finite‐element methods (FEM; Comsol Multipysics). In broadband frequencies, effective shear‐type response is well excited as designed (see **Figure** **3**a and Figure S5 in the Supporting Information). Figure 3a displays the modal response in steady and resonant states. For steady frequencies, dual drive signals lead to more ideal shear‐like deformation because of their diagonally coordinate actions of all subunits. Perfect face‐shear deformation is calculated to appear at resonant frequencies for both dual drive and single drive signals (at around 80.3 kHz). To validate the simulated predication, Figure 3b experimentally measures the impedance spectra of prepared sample under single drive signals, and a consistent resonance peak around 85.1 kHz is detected.

Figure 3c compares the displacement outputs of conventional d_15_, d_24_ bulk structure and quasi‐d_36_ single‐layer or cofired 7‐layer structure of the same geometrical dimensions (see calculation details and analytical solution in Section S3 of the Supporting Information). Under equal drive voltages, numerical calculation demonstrates that the cofired one produces remarkably hugest outputs, whose values are bigger than those of natural mode by over one order of magnitude. The giant response arises from two factors: that quasi‐d_36_ face shear deformation occurs in the main geometrical plane with big effective transverse line element similar to d_24_ mode and, more importantly, both electric fields along thickness direction and multilayer construction will sharply decrease working voltage. The displacement variation tendency along with applied electric fields, especially for single drive way, is further discussed (Figure S6, Supporting Information). When considering the key motion point for actuation, single drive method can produce comparable outputs to dual ones and, notably, is very meaningful in practical engineering. Since external electric fields and polarization are totally along with the same direction, a quite higher fields can be offered to supply bigger displacements and the technical deficiency of domain switching problem at high electric fields in traditional d_15_ and d_24_ mode can be effectively avoided.

The colossal piezoelectric response of the structure including charge generation and displacement outputs are then experimentally verified. Small signal piezoelectric charge response of the cofired specimen is measured by classic quasi‐static method with aid of a specially made adaptor (Figure 3d).^[^ ^44^ ^]^ With one pair side surfaces sheared by parallel loads, a displayed value of 3156 pC N^−1^ is observed. Usually, there should exist no charge response on “3” main plane under pure T_6_ shear force excitation because of the microscopically symmetry of poled piezoceramics. However, similar to a lot of piezoelectric elements with asymmetrically mechanical boundary conditions in actual devices, the one pair fixed surfaces are restricted to freely deform under applied loads, so that the imperfect shear forces will result in distributed normal stress and additional contraction or extension distortion (d_31_ effect) and finally, lead to charge release (Figure S7, Supporting Information). The displayed value of cofired sample is about 4.5 times bigger than that of d_36_ single‐layer one (710 pC N^−1^). Although it should be noted that the charge outputs are the synthetic effects of d_31_ mode in essence and also related to other factors such force points, the specimen with shear force‐induced stable charge is qualified to behave as the sensitive cell in piezoelectrical sensors. Notably, the measured bigger shear‐type charge response of cofired sample indicates heightened sensing capability, which derives from its multilayer structure.

**Figure 4:**
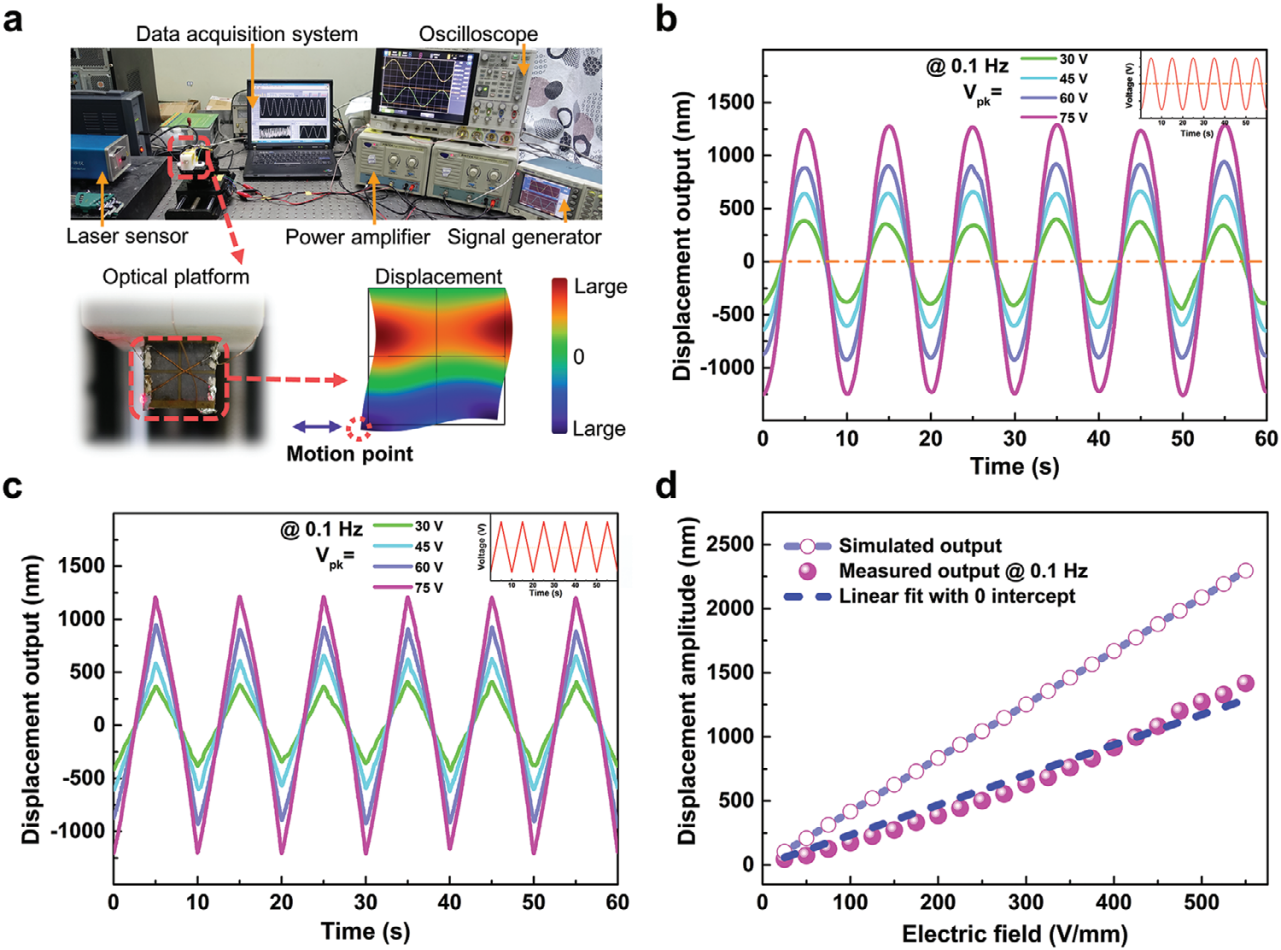
Experimental measurements of actuation performance of cofired 7‐layer specimen under dual drive signals. a) Schematic diagram of measurement system composed of a drive module and a nanometer resolution displacement measuring module. b,c) Real‐time displacement response of the prototypical actuator at 0.1 Hz under various b) sinusoidal and c) zigzag driving signals. d) Measured displacement amplitude is basically linear to applied electric fields and corresponds to simulated results.

To investigate the actuation performance, displacement response of the specimen is experimentally detected. **Figure** **4**a clarifies the measurement system. One side surface of the 7‐layer prototype actuator is fixed on an optical platform, and a vertex of its opposite side is regarded as motion point for quantifying effective shear‐type outputs. When AC dual drive signals is offered, its transverse displacement is characterized by a nanometer‐resolution laser feedback interferometer and recorded automatically by a data acquisition system. Figure 4b,c presents the real‐time displacement answers of the actuator at representative 0.1 Hz sinusoidal and zigzag drive signals with various voltages, where stable and repeatable displacements are well generated. A basically linear response to zigzag signals reveals good linearity and high‐level controllability for actuation. Measured displacement amplitudes as a function of electric fields are plotted and fitted in Figure 4d, and the curve presents a positively linear relationship, which also agrees with the simulated predications. More importantly, a giant displacement output as high as ±1.25 µm can be easily obtained with drive voltage of only ±75 V, which is 22.5 times bigger than natural d_15_ mode actuator with the same geometrical dimensions and drive voltage (theoretical maximum value is ±55.5 nm; *d*_15_ = 740 pm V^−1^). This proves that shear‐type actuators can also output considerable displacement without any complex amplification mechanism. The measured values are reasonably inferior to simulated ones because of factors such as normal cofired degradation, locally imperfect polarization, inoperative piezoceramic boundary areas, and relatively simplified simulation model.

We demonstrate that the neotype multilayer cofired shear‐mode device can effectively produce shear‐type outputs at broad frequencies and low drive voltages. In fact, the presented performance can easily be much further optimized by dozens of times through advanced industrialized‐level MLCC technology. Based on our structure, the drive voltage should be decreased to ultralow level (below ten volts) by a few micros thick layer, the device sizes are supposed to be controlled below millimeter, and also, robust mechanical and electrical reliability can be guaranteed.

### 2.3 Enhanced Performance for Shear‐type Actuators and Sensors

According to the measurements and analyses above, this novel design firstly brings in practical cofired multilayer shear mode devices to piezoelectricity. Besides high integration, low cost and compatibility with advanced microelectronic technologies, the novel structure should present two main obvious advantages: 1) to generate considerable displacements in the situation of low drive voltages for actuation; 2) to produce enhanced charge under the same strain for enlarging sensing capabilities. Here, to validate the service performance, we experimentally investigate two typical applications.

Besides primary actuation,^[^ ^16^ ^]^ traditional piezoelectric d_36_ face‐shear elements are widely used in stepper or ultrasonic motors,^[^ ^15^ ^]^ sensors^[^ ^18^, ^45^ ^]^ and guided‐wave nondestructive transducers.^[^ ^20^, ^21^, ^41^, ^46^ ^]^ The real‐time structure health monitoring (SHM) of widespread large plates such as oil pipelines is very challenging in modern industries. And guided fundamental shear horizonal wave (SH_0_) based inspection technology by distributed shear‐mode piezoelectric elements is reported to be one of the most preponderant method for SHM with high energy conversion efficiency and nondispersive characteristic.^[^ ^20^, ^47^ ^]^ Herein, the cofired 7‐layer specimen is adopted as excitation and reception elements of SH_0_ wave to examine their actuation and sensing performance.

**Figure 5:**
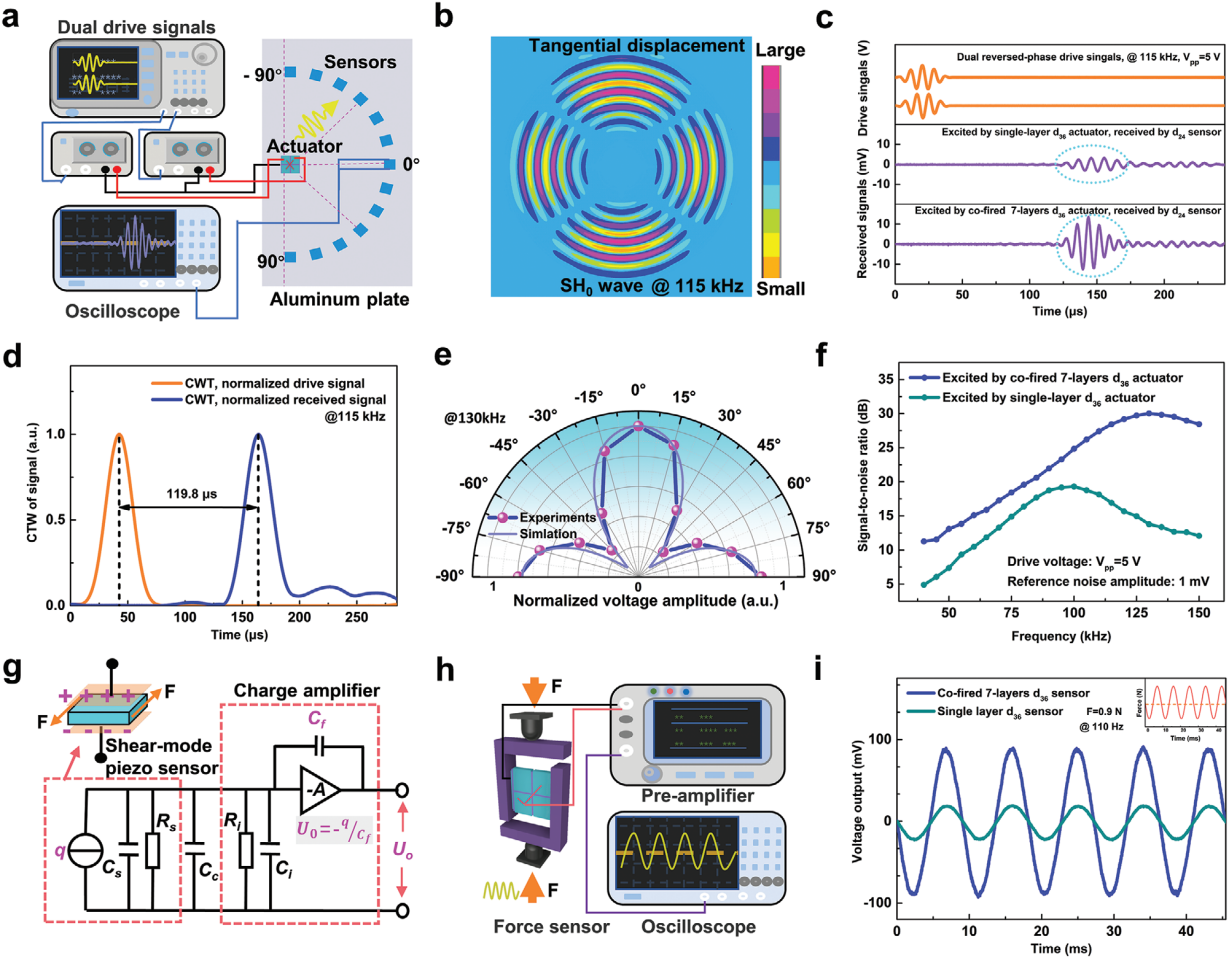
For shear‐mode applications as actuators or sensors, enhanced performance of the cofired 7‐layer specimen is experimentally investigated and demonstrated. a) Test system of excitation and reception of SH_0_ wave for SHM, where the cofired d_36_ sample and conventional d_24_ samples behave as actuators or sensors in turn. b) Finite element simulation results of SH_0_ wave generated by cofired sample. c) Wave signals excited by cofired sample and received along the principal direction, and d) the time domain signals by CWT at 115 kHz. e) Received signals as a function of spatial angles at 130 kHz and f) working frequencies. g) The charge detection way for piezoelectric sensors, where voltage outputs are proportional to original tiny charge outputs (see symbol details in Figure S10 in the Supporting Information). h) Driven by sinusoidal force, the charge signals are transformed to voltage outputs and detected, and i) the cofired multilayer specimen contributes to much enhanced response compared with single layer one.

**Figure** **5**a schematically shows the testing systems, where the key geometrical dimensions can be found in Figure S8 in the Supporting Information. A large aluminum plate is used as guided‐wave media, and the cofired 7‐layer specimen behaves as an actuator to emit SH_0_ wave, which is driven by dual inverse‐phase signals of five‐cycle sinusoid tone‐burst enclosed in Hanning window. Circular‐distributed d_24_ mode piezoceramic sensors with 15° gap angles will receive and quantify the signal strength. Time‐transient FEM simulation (ANSYS code) is initially conducted to examine the capabilities of the artificial cofired sample in generating and receiving SH_0_ wave (Figure S8, Supporting Information). And Figure 5b denotes that SH_0_ wave should be well excited with the maximum amplitudes along the main axes (0° and ± 90°). To experimentally investigate the actual excitation effects of cofired specimen, a single‐layer quasi‐d_36_ actuator is adopted as contrast. Under 5 volts peak‐peak drive signals of a central frequency at 115 kHz, the received wave package along 0° degree is observed to be enhanced by 3 times when driven by the cofired actuator (Figure 5c). This is because that at the same driving voltage, multilayer specimen will lead to bigger electric fields and corresponding larger strain. Continuous wavelet transform (CWT) based on complex Morelet wavelet is used to analyze signal components (Figure 5d). A time interval of 119.8 µs between the excitation and reception signals appears in the time domain, and the group velocity of the wave package is calculated to be 3005 m s^−1^, which agrees well with the theoretical velocity of 3099 m s^−1^ and validates pure SH_0_ wave. Spatial angle response at 130 kHz is measured in Figure 5e. It is shown that the signal strength is dyad‐symmetric and the strongest response emerges in the main axes, just like results in Figure 5b. Frequency‐dependent answers display in Figure 5f. Although voltage outputs are also related to the frequency characteristic of d_24_ sensors, it is clearly shown that in broadband range, signal‐to‐noise ratio (SNR) is much enhanced when driven by the cofired multilayer structure.

Behaving as a sensor, the reception voltages is directly tested and expounded and the cofired structure also performs well over wide bands (Figure S9, Supporting Information). In fact, for piezoelectric sensors, charge‐detection method assisted with charge preamplifiers is more effective, robust and is the mainstream technical route especially for tiny signals, where output strength is proportional to generated charge instead of voltage of sensors^[^ ^48^, ^49^ ^]^ (see principles in Figure 5g and Figure S10 of the Supporting Information). Since multilayer structure can greatly amplify charge response, cofired devices should heighten sensing capabilities. Here we demonstrate a simple prototypical charge‐based force transducer. The same sinusoidal drive loads are applied to the cofired 7‐layer and single‐layer quasi‐d_36_ sensors in turn, and their charge signals are preamplified and detected (Figure 5h). The voltage outputs in Figure 5i clearly reveal that the cofired multilayer sensor leads to much bigger response as theoretical predication. With abilities to sharply decrease the drive voltage of actuation components and enhance charge response of sensing units, the innovative cofired multilayer structure is expected to effectively renovate shear‐mode piezoelectric devices, such as nano shear actuators,^[^ ^22^ ^]^ quasi‐static or resonant ultrasonic motors,^[^ ^15^ ^]^ accelerometer sensor,^[^ ^19^ ^]^ etc.

## 3 Conclusion and Discussion

One of the most significant, pressing, and long‐term challenges in the field of piezoelectric technologies and devices concerns how to realize advanced cofired preparation of basic shear‐mode multilayer structure. The biggest stumbling block to this is that cofired technologies inevitably require parallel electric fields and polarization, which is incompatible with all naturally occurring piezoceramic shear modes. Learning from the principles of metamaterials, we design, fabricate, and first bring a prototypical cofired shear‐mode 7‐layer structure into piezoelectricity. These results should fundamentally boost the development of shear‐type piezoelectric technologies.

From the engineering point of view, our design promises to wipe out the practical mass production barriers for integrated shear‐type multilayer piezoelectric devices in microelectronics industry. Besides, the design is demonstrated to be able to very effectively enhance the output performance of both shear‐type actuators and sensors.

Theoretically speaking, the methodology expands the basic understandings of building piezoelectric devices and even other systems with multiphysical coupling nature. It reveals that the modal excitation may not only be restricted to traditional methods such as changing drive frequencies or adjusting classical structures based on naturally existing physical parameters. The generalized design physiology of metamaterials is also a very powerful means to learn from (such as results in this work). By constructing geometrical and topological structures with arrayed basic function subunits (meta‐atoms) and utilizing or manipulating their synergistic effects, naturally nonexistent effects and devices are expected to be created.

## 4 Experimental Section

##### Cofired Shear Mode Multilayer Specimen Fabrication

Low temperature cofiring PZT‐5H powder (Xi'an Kanghong piezoelectric technology corporation) was ball‐milled for 12 h with polyvinyl alcohol as binder, di‐*n*‐octyl *ortho*‐phthalate as plasticizer and ethyl acetate as solvent to form functional precursor slurry. Embryo thick films were prepared by tape casting technology (AFA‐3, MTI corporation), dried, and cut into smaller pieces with screen‐printed patterned electrodes. During lamination, the stacked thin pieces were molded into one compact unibody by hot‐isostatic‐pressing (HIP) under pressure of 200 MPa at 100 °C for 20 min. After organic ingredients burned off at 600 °C, the green compact was buried in PZT‐5H powder and sintered in muffle at 950 °C for 3 h. The obtained specimen was polished to get exposed inner electrodes and flat side surfaces. Then external electrodes were screen‐printed onto the side surfaces with a postfire at 650 °C for 0.5 h to achieve well electrically parallel connection. Specimens were poled in silicone oil bath under a DC electric field of 30 kV mm^−1^ in 120 °C for 15 min, whose subunits in the same layer are poled along the same direction.

##### Characterization of Physical and Electrical Properties

The microscopic morphologies of obtained specimen were observed by SEM (Zeiss Merlin Compact). The phase and crystallization structure were determined by XRD method with Cu K*α* radiation (PANalytical X‐Pert3 Powder). Piezoelectric coefficient *d*_33_ and piezoelectric charge response of the cofired specimen were measured using a quasi‐static *d*_33_ meter (ZJ‐3D, Institute of Acoustics, Beijing, China). The polarization–electric field hysteresis loops and the field‐induced strain curves were measured by a ferroelectric testing system (aixACCT TF Analyzer 1000, aixACCT, Aachen, Germany). The capacitance and dielectric properties were measured by a precisely impedance analyzer assisted by a programmable muffle (4294A, Agilent Technologies). Since the analyzer can only provide one channel, the experimental resonance and antiresonance spectrum was measured in situation of single drive way.

##### Finite Element Analyses

Deformation states of the cofired 7‐layer structure along with frequencies, and effective displacement outputs of all modes were simulated by COMSOL code in piezoelectric module. The geometrical models of cofired specimen were simplified, where the small piezoceramic boundary regions between four subunits and at the edges were reasonably disregarded. Default PZT‐5H materials were selected from the material libraries and separately polarized by establishing rotated coordinate systems. With appropriate fixed mechanical boundary conditions and very detailed mesh generated by physical fields, finite element analyses were conducted. Frequency response module was used to simulate the deformation actions and calculated impedance spectrum. To calculate displacement outputs and corresponding variation tendency with applied voltage or electric fields, one side perpendicular to main shear surface of all modes was mechanically fixed, and the vertex of opposite side was adopted as effective motion point.

The capabilities and performance of cofired 7‐layer *d*_36_ shear mode structure in excitation and reception of SH_0_ wave were analyzed using ANSYS code. An aluminum plate with 2 mm thickness was modeled by Solid 185 module, where its density, Young's modulus and Poisson ratio were 2700 kg m^−3^, 69 GPa and 0.3, respectively. All PZT‐5H specimens including cofired one were modeled by Solid 5 and separately bonded on the plate as design. A five‐cycle Hanning window‐modulated sinusoid toneburst was used as excitation signal to drive piezoelectric actuator and induce mechanically guided‐wave in aluminum media. The received voltage signal of piezoelectric elements was also calculated. Detailed simulation results are explicated in Figure S8 in the Supporting Information.

##### Experimental Verification of Actuation and Sensing Applications

The basic actuation performance of the cofired 7‐layer specimen was measured by electric field induced displacement method. The prototype actuator was fixed onto 3D printed fixture with one side surface fixed. Dual drive signals with inverse phase were provided (Tektronix AFG3022B or Agilent 33522A), amplified (PINTEK HA‐405), and monitored (Keysight MSOX4024A). The displacement outputs were measured by a high precision laser feedback interferometer (LeiCe LY1000) and recorded by a data acquisition system (LabVIEW). To examine the performance of the cofired specimen in generating SH_0_ wave for SHM, an aluminum plate with dimensions 1000 mm × 1000 mm × 2 mm was used as substrate guided‐wave media. Arrays of d_24_ mode PZT‐5H specimens with sizes of 6 mm × 6 mm × 1 mm were adopted as sensors. Then for measuring reception performance of cofired specimen, d_24_ mode specimens behaved as actuators. To examine the performance of the cofired specimen as force transducer, the applied loads were provided by quasi‐static d_33_ meter and calibrated by commercialized piezoelectric force sensor. The charge outputs were amplified by a charge amplifier (Kistler 5015) and detected by an oscilloscope.

## Conflict of Interest

The authors declare no conflict of interest.

## Author Contributions

The project was conceived and designed by J.Y. under guidance of S.D.; J.Y. designed and prepared cofired shear mode multilayer samples, performed the simulation and experiments, and analyzed the data; Q.H. and F.L. helped in guided wave application and simulation. Y.Y. assisted in fabrication of cofired samples; J.W., Z.C., and M.P. assisted in measurement of electric properties. The manuscript was prepared and written by J.Y.

## Supporting information

Supporting Information
