# Supplementary material for: Tailoring Artificial Mode to Enable Cofired Integration of Shear‐type Piezoelectric Devices
Source: Adv Sci (Weinh). 2020 Jul 6;7(17):2001368. doi: 10.1002/advs.202001368 (PMC7507555; doi:10.1002/advs.202001368)
Supplement: Supplementary file 1 — Supporting Information [file ADVS-7-2001368-s001.pdf]

Supporting Information

Tailoring Artificial Mode to Enable Co-fired Integration of Shear-type Piezoelectric Devices

*Jikun Yang[1,3], Qiang Huan[2], Yang Yu[1], Jingen Wu[1], Zhaoqiang Chu[1], Mohammad Javad Pourhosseini Asl[1], Faxin Li[2] and Shuxiang Dong[1, 3, *]*

[1] *Department of Materials Science and Engineering, College of Engineering, Peking University, Beijing 100871, China*

[2] *LTCS and Department of Mechanics and Engineering Science, College of Engineering, Peking University, Beijing 100871, China*

[3] *Beijing Key Laboratory for Magnetoelectric Materials and Devices (BKL-MEMD), Peking University, Beijing 100871, China*

[*] *Corresponding e-mail: sxdong@pku.edu.cn*

Contents

Section S1. Theoretical analysis of exciting quasi-d_{36} shear mode.

Section S2. Basic characterization of the co-fired specimen.

Section S3. Calculation and comparation of displacement outputs.

Section S4. Charge generation studies.

Section S5. Other simulated and experimental results of SHM.

Section S6. Theoretical analysis of enhanced sensing capabilities.

Section S1. Theoretical analysis of exciting quasi−d_{36} shear mode

Naturally occurring shear modes of piezoceramics include in-plane poled d_{15} (Figure 1a), thickness poled d_{15} (**Figure S1**a) and in-plane poled d_{24} modes (Figure S1b). Due to various electrical and mechanical boundary conditions, d_{15} modes and d_{24} mode occur in the side surfaces and main surface of piezoelectric elements, respectively. It should be noted that applied electric fields along with thickness direction (t) instead of length direction (l, l is usually quite a lot bigger than t) will effectively decrease working voltages of piezoelectric elements. For example, in-plane poled d_{15} mode requires smaller drive voltage compared with thickness poled d_{15} mode to generate the same displacement. On the other hand, in-plane face shear deformation such as d_{24} mode instead of side shear deformation will contribute to bigger displacement outputs in the situation of the same electric fields, which derives from the longer effective line elements l. To conclude, shear mode that happens in the main surface of specimen with bigger edge length l and is driven by electric fields along thickness direction with smaller thickness t would be much more efficient for actuation applications at the same voltage. Naturally occurring d_{36} face shear mode in some single crystals meet the both features. More importantly, d_{36} mode is totally compatible with co-fired technologies to realize shear-mode multilayer structure. Here, theoretical analyses will reveal the viability of our method to excite quasi-d_{36} shear mode.

To create artificial d_{36} mode of piezoceramics, four subunits (for single-layer or multilayer structures) poled along the same “3” direction are applied diagonally anisotropic electric fields (as illustrated in Figure 1b). Each subunit actually works on d_{31} normal-strain mode, and their combined effect will result in one extensional and one contractional diagonals, as shown in Figure S1c and d. For a square-shaped specimen, when 1′ and 2′ axes along with diagonals are taken as the prime coordinates, stress tensor in this situation can be simplified as

$$S' = \begin{bmatrix} -s & 0 & 0 \\ 0 & s & 0 \\ 0 & 0 & 0 \end{bmatrix} \quad \text{(Equation S1)}$$

where s and -s means extensional and contractional stresses. When 1 and 2 axes along with the specimen’s sides are regarded as principal axes, by using transformation matrix

$$T = \begin{bmatrix} cos\theta & sin\theta & 0 \\ -sin\theta & cos\theta & 0 \\ 0 & 0 & 1 \end{bmatrix} \text{ (where } \theta\text{=-45°)} \quad \text{(Equation S2)}$$

its equivalent stress tensor can be calculated as

$$S = \mathrm{T}S'\mathrm{T}^{-1} = \begin{bmatrix} 0 & -s & 0 \\ -s & 0 & 0 \\ 0 & 0 & 0 \end{bmatrix} = -\mathrm{S}_6 \quad \text{(Equation S3)}$$

The components S_{12} and S_{21} have the same values s, which means that a pure shear stress S_6 is generated equivalently. So quasi-d_{36} mode is demonstrated to be successfully generated by our design.

When we consider the single drive way shown in Figure 3 in the main manuscript, the stress tensor in (1′2′3) coordinates presents as

$$S' = \begin{bmatrix} -s & 0 & 0 \\ 0 & 0 & 0 \\ 0 & 0 & 0 \end{bmatrix} \quad \text{(Equation S4)}$$

and the transformed equivalent stress tensor S in (123) coordinates is

$$S = -\frac{1}{2}\begin{bmatrix} s & s & 0 \\ s & s & 0 \\ 0 & 0 & 0 \end{bmatrix} = -\frac{1}{2}\begin{bmatrix} 0 & s & 0 \\ s & 0 & 0 \\ 0 & 0 & 0 \end{bmatrix} - \frac{1}{2}\begin{bmatrix} s & 0 & 0 \\ 0 & s & 0 \\ 0 & 0 & 0 \end{bmatrix} = -\frac{1}{2}S_6 - \frac{1}{2}(S_1+S_2) \qquad \text{(Equation S5)}$$

The stress tensor is composed of two items: The first item is equivalently shear stress S_6, and the second item derives from additional contractional stresses. This solution reveals single drive way can also lead to shear-type deformation, just with some contractional deformation, and the results are consistent with FEM simulation results in Figure 3 and Figure S5. When we consider the motion point for actuation applications, a comparable output to that of dual drive ways will still be obtained.

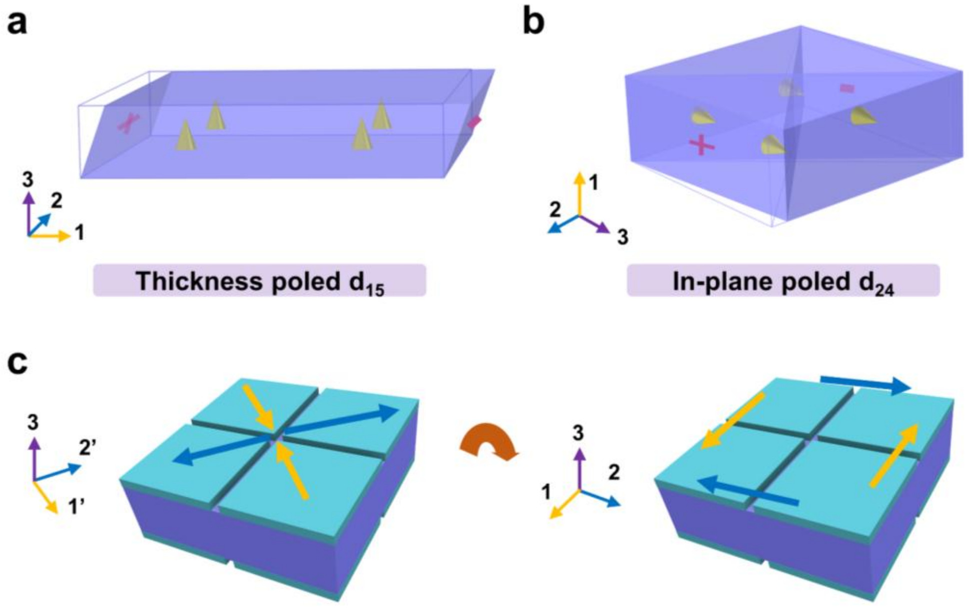

Figure S1. Schematic diagrams of thickness poled d_{15}, in-plane poled d_{24} mode and stress analyses of quasi-d_{36} mode. **a**) Naturally occurring thickness poled d_{15} shear mode, which occurs in side surface and usually requires higher applied voltage to generate the same displacement of in-plane poled d_{15} shear strain mode. **b**) Naturally occurring in-plane poled d_{24} shear mode, which occurs in the main plane of piezoelectric specimen. **c, d)** The extensional and contractional stresses along diagonals of d_{36} structure (c) can be demonstrated to be equivalent to mechanical conditions of shear stresses (d).

Section S2. Basic characterization of the co-fired specimen

To verify the fabrication qualities and guarantee the effective outputs of the co-fired specimen, the basic physical properties of its subunits are investigated in detail. **Figure S2** expounds on the fundamental piezoelectric and ferroelectric properties of raw low temperature sintered PZT-5H materials.

The geometrical dimensions, phase structures, microscopic morphologies and piezoceramic element distribution of co-fired specimen are shown in **Figure S3**. Fine crystallization of a fresh cross section in a wide range is detected by SEM method. The element distribution of ceramic layers on the cross section is also detected by EDS method.

Figure S4 tests the basic electromechanical and dielectric response of a square quarter of co-fired 7-layers d_{36} specimen. The edge length of the quarter part is 6.5 mm, and the total thickness is 1.05 mm. Since quasi-d_{36} deformation derives from the normal strain deformation of each single subunit, the tested giant effective d_{33}^{*} will guarantee effective transverse displacements of synthetic d_{36} mode. Based on capacitance measurements, the temperature and frequency dependence of dielectric permittivity can be calculated and dielectric behaviors of co-fired specimen are revealed. Dielectric constant is calculated by measuring capacitance ($\varepsilon_r = {Ct}/{\varepsilon_0 S n^2}$, where ε_r is relative dielectric constant, ε_0 is the permittivity of vacuum, *C* is measured capacitance; *t* is the total thickness; *S* is the plate area; *n* is the layer number). In our measurement, the purpose is to detect the Curie temperature and the dielectric variation tendency, so we assume that the electrode area of each layer is the same large as that of the ceramic layers to simplify calculation. But actually, the inner interdigital electrodes of that quarter sample don't cover all surfaces of ceramic layers, so the dielectric constant of co-fired sample is slightly underestimated.

All these results of comparison measurements ensure that every inner functional layer of co-fired specimen is electrically connected out and functions as well as pure bulk wafer.

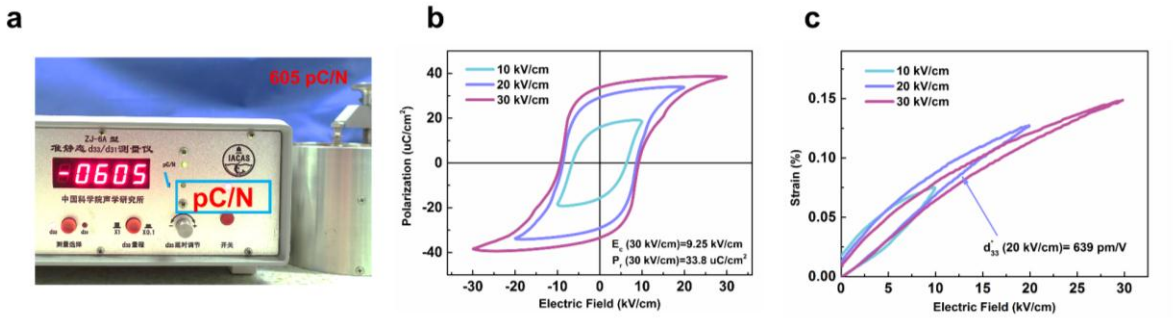

Figure S2. Basic properties of commercial raw PZT-5H ceramics for co-fired devices. The raw material has been doped to decrease its sintering temperature to 950 ℃ for co-sintered with silver electrodes. **a**) Piezoelectric d_{33} coefficient is measured to be 605 pC/N. **b**, **c**) Ferroelectric hysteresis loops (b) and unipolar strain curves (c) of PZT-5H ceramics are measured based on a circular wafer sample (diameter: 11 mm; thickness: 1 mm). Coercive electric field (E_c) and remanent polarization (P_r) are tested to be 9.25 kV/cm and 33.8 μm/cm^2, respectively. From the strain curves, big signal d_{33}^{*} is measured as 639 pm/V.

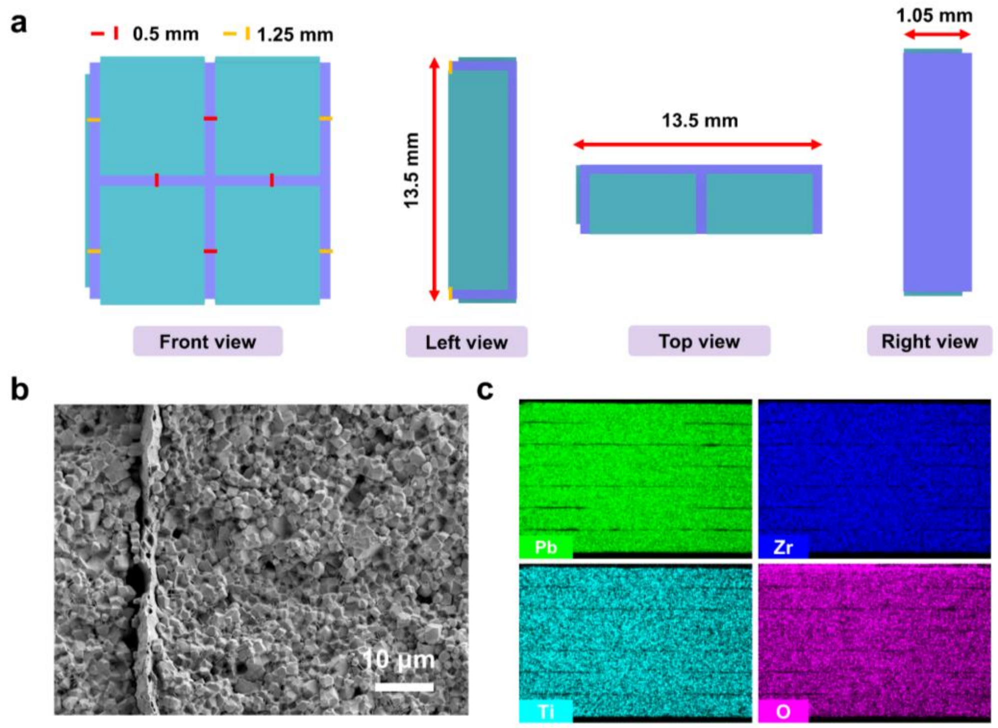

Figure S3. Geometrical dimensions, phase, morphology and composition verification of the co-fired sample. **a**, Detailed structure and geometrical dimensions of prepared specimen. **b**, SEM result of fresh cross section of the multilayer device, and the fine crystallization in a wide range is guaranteed for good piezoelectric property. **c**, EDS results of piezoceramic layers also show flat interfaces and ideal element distribution as designed.

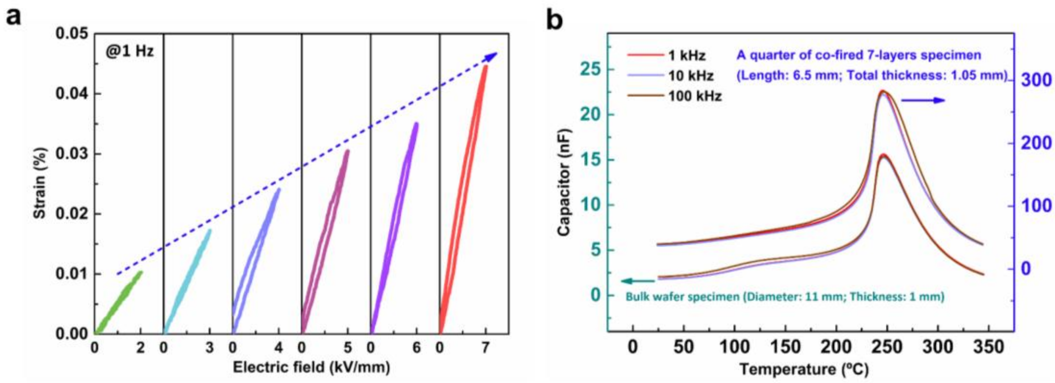

Figure S4. Measurements of basic electromechanical and dielectric properties of a square quarter of co-fired specimen. **a**) Real-time strain response to applied electric fields ranging from 2 to 7 KV/cm is tested. **b**) temperature dependence of measured capacitors of a quarter of the co-fired sample and a single-layer wafer for comparison.

Section S3. Calculation and comparation of displacement outputs

Here we show the finite element simulation and theoretical analyses of deformation states and displacement outputs of the co-fired d_{36} multilayer and other shear mode structures. **Figure S5** schematically exhibits the simulated face shear deformation of co-fired 7-layers structure at broadband frequencies under dual drive signals and single drive signal. In a wide range away from resonant frequency, displacement outputs are stable. When excitation frequencies get close to resonant states, the deformation becomes more ideal and displacement turns bigger.

Figure S6 gives the details to calculate displacement output capabilities of all naturally occurring shear modes and artificial single-layer or co-fired 7-layers quasi-d_{36} mode of the same geometrical dimensions (with length l and thickness t). With boundary conditions of fixed surfaces perpendicular to the planes where shear deformation occurs, the variation tendency of displacement of key motion points along with drive voltages and drive electric fields are calculated by FEM method (Figure S6a). Due to the two advantages expounded in Section S1, quasi-d_{36} mode will generate much bigger outputs than other modes at the same voltage as shown in Figure 3c. Theoretically analytic expressions of quantitative displacement D^{eff} of naturally occurring modes can be given as

$$D^{eff}_{In-plane\ poled\ d_{15}} = d_{15}E_1 t = d_{15}V \qquad \text{(Equation S6)}$$

$$D^{eff}_{Thickness\ poled\ d_{15}} = d_{15}E_1 t = \frac{d_{15}Vt}{l} \qquad \text{(Equation S7)}$$

$$D^{eff}_{In-plane\ poled\ d_{24}} = d_{24}E_2 l = d_{24}V \qquad \text{(Equation S8)}$$

where l and t are the length and thickness of piezoelectric elements, respectively. E and V denote drive electric fields and drive voltages. It should be noted that the same subscripts of E and V of various modes do not mean the same directions because they belong to different coordinate systems (to keep "3" as default polarization direction). And the coefficient d_{15} physically equals to d_{24}.

As the analyses in Section S1, quasi-d_{36} face shear mode can be regarded as the synthetic action of four d_{31} mode subunits, and its displacement should be proportional to the deformation of each subunits. In accordance with simulation results, the displacement D^{eff} of artificial d_{36} mode presents as

$$D^{eff}_{Single\ layer\ d_{36}} = \alpha d_{31}E_3 l = \frac{\alpha d_{31}Vl}{t} \qquad \text{(Equation S9)}$$

$$D^{eff}_{Co-fired\ multilayer\ d_{36}} = \alpha d_{31}E_3 l = \frac{N\alpha d_{31}Vl}{t} \qquad \text{(Equation S10)}$$

where t means the total thickness of piezoelectric elements, and N is number of layers. Parameter α represents the scale factor and is related to geometrical dimensions of specimens. The exact value of α is hard to obtain because of the nonlinearly contractional or extensional deformation derived from the stress concentration at the borders of four subunits. However, further simulation results and analysis indicate that α should be bigger than 0.25 and less than 0.5 in a wide range.

Since length l is usually much bigger than thickness t (for example, fabricated multilayer specimen in this work is 13.5 mm long and 1.05 mm thick), according to the formulas above, D^{eff} of single layer d_{36} mode should be much bigger than naturally d_{15} or d_{24} modes at the same drive voltage. Further, the co-fired N-

layers d_{36} mode structure should generates N times bigger displacement than single-layer one at the same drive voltage. These analytic results are consistent well with the variation tendency calculated by FEM (Figure S6b).

Figure S6c investigates the advantages of single drive way of co-fired multilayer d_{36} structure: much bigger external electric fields can be applied to generate bigger displacement output without problems of domain switching and service failure. For shear-strain d_{24}/d_{15} modes or contractional normal-strain d_{33} mode, the working electric fields are usually limited to 1/2 coercive electric field (Ec). However, for extensional normal-strain d_{33} mode, much higher working electric fields can be offered (as long as they are smaller than the breakdown value, and the breakdown value is usually 2 to 3 or higher times bigger than Ec). Since applied electric fields are totally in the same direction of polarization in the condition that d_{36} mode is excited by single drive signal, the structure can withstand much bigger electric fields and contributes to further enhanced displacements. According to commercialized d_{33} multilayer devices, their tolerant limits of electric fields are about 2 to 5 times bigger than normal d_{15} mode. Besides, this interesting feature should also guarantee the service reliability by reducing the risk of domain switching.

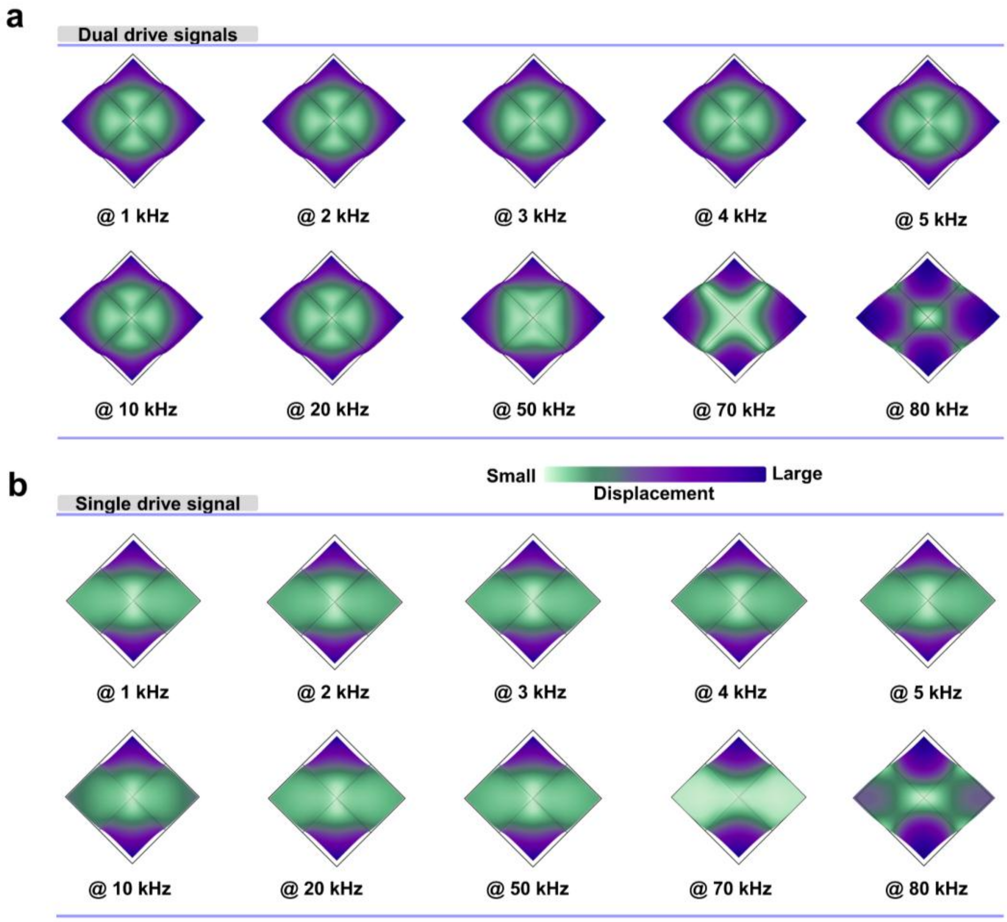

Figure S5. Frequency dependence of simulated deformation of co-fired d_{36} structure under dual or single drive ways. **a**, **b**) In wide frequencies, objective shear-mode deformation is successfully excited by dual drive signals (**a**) and single drive signal (**b**). When drive frequency gets close to resonant one, shear deformation turns bigger and more ideal. This is due to the equipartition of energy in space.

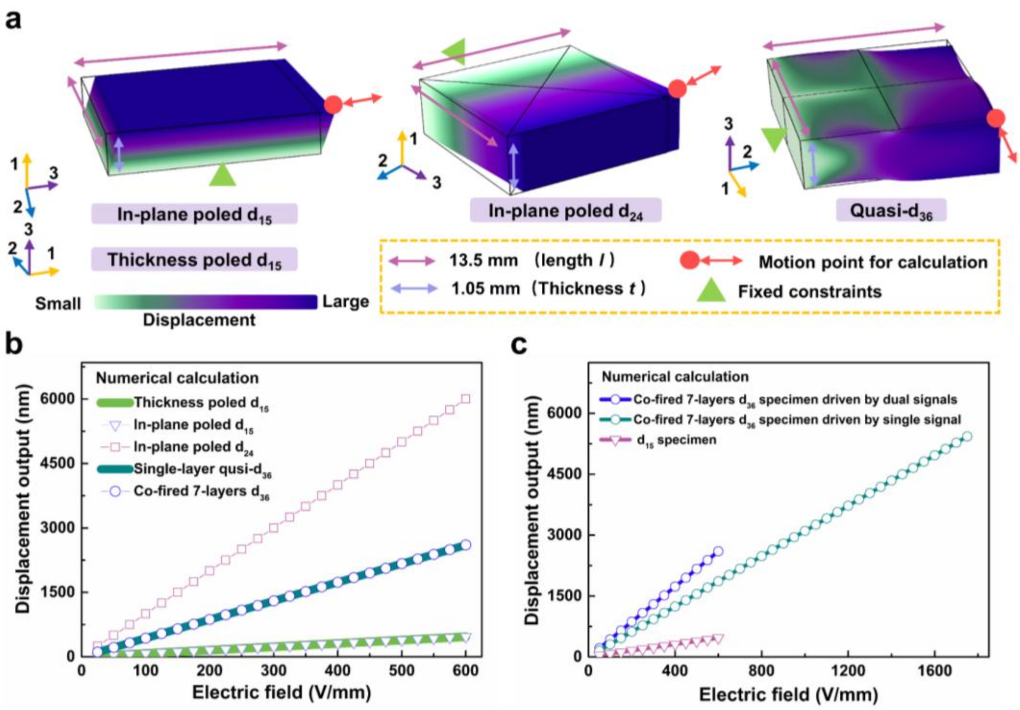

Figure S6. Calculated details and comparation for displacement outputs of various shear-type modes. **a**) Geometrical models, boundary conditions, deformation states and motion points for calculating effective displacement outputs. **b**) Under the same drive electric fields, co-fired structure shows the same displacements as single-layer d_{36} structure, which is smaller than d_{24} mode but is still much bigger than d_{15} mode. **c**) With applied electric fields and polarization totally along the same direction, single drive way can tolerate much higher electric fields and contribute to big displacements without domain switching problems.

Section S4. Charge generation studies

As analyzed in the main manuscript, there should not exist charge outputs on planes along with "3" direction if the co-fired quasi-d_{36} 7-layers specimen is sheared by perfectly pure T_6 shear force (**Figure S7**a). While in practical sensing applications, devices based on this d_{36} structure can also be designed to generate equivalently charge signals by the aid of contractional or extensional deformation (equivalently piezoelectric charge coefficient d_{36}), just as the inverse piezoelectric displacement outputs where four subunits working on d_{31} modes contribute to synthetic d_{36} deformation. Actually, the charge response detected by quasi-static method is a similar example.

The detailed force analysis during the measurements is schematically shown in Figure S7b. When stable external loads are applied, the fixed boundary conditions will restrict uniform deformation, and further induce additional contractional or extensional effects of the co-fired specimen, so that stable and repeatable charge will be generated in the main "3" planes. Figure S7c and d exhibit the charge response of single-layer d_{36} specimen and co-fired 7-layers specimen, respectively. It is obvious that the multilayer structure will strongly amplify charge response. This enhancement is very meaningful in charge-based piezoelectric sensors.

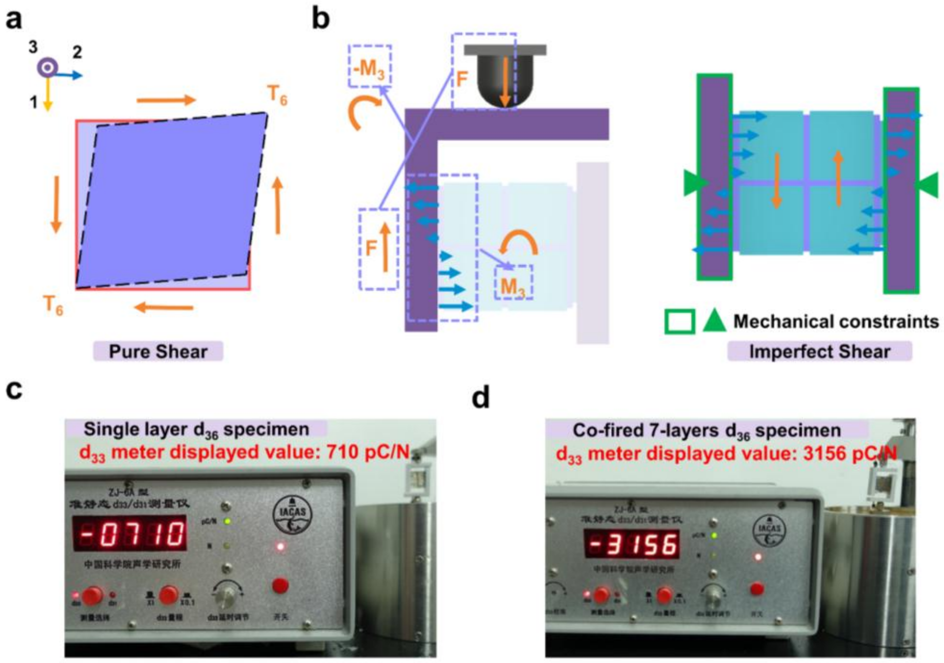

Figure S7. Force analysis and charge generation capability of co-fired d_{36} specimen measured by quasi-static method. **a**) Pure shear deformation should by driven by pure shear force T_6 (T_{12} or T_{21}), where non contraction or extension deformation exists, and no charge will be generated in surface "3". **b**) When specimen is tested by home-made adaptors based on quasi-static method, besides equivalent shear stress, the stress distribution and unsymmetrical fixed boundary conditions will cause additively contractional and extensional deformation, and induce charge response in surface "3". **c**, **d**) Charge response of sing-layer d_{36} and co-fired 7-layers d_{36} specimens of the same dimensions and testing condition.

Section S5. Other simulated and experimental results of SHM

Here we show the detailed simulation studies on generation and reception of SH_0 waves (**Figure S8**) and experimental SH_0 reception performance of co-fired d_{36} 7-layers specimen **(Figure S9**) for structural health monitoring (SHM) applications.

Dispersion problems exist in most guided-wave modes and will result in distorted signals and tough signal processing problems in SHM applications. Based on the Navier's displacement equation, SH_0 wavers in thin plates can be calculated to feature stable group velocity irrelevant to drive frequencies (Figure S8b). The wave-fields generated by co-fired d_{36} specimen are studied by FEM simulation. In the cylindrical coordinates, the simulated tangential displacement component, radial displacement component and out-of-plane component stand for SH_0, S_0 and A_0 wave modes, respectively. It is shown that SH_0 and Lamb waves (A_0 and S_0 waves) all happen as predicated (Figure 5b; Figure S8 c,d). To investigate the generation and reception capabilities of co-fired d_{36} 7-layers structure, the tangential displacement signal excited by quasi-d_{36} specimen and voltage response signal received by quasi-d_{36} specimen at the principal axis (0°) are simulated (Angle 0° has minimal S_0 and A_0 interferences), respectively. It is shown that quasi-d_{36} specimen works well in generation and reception of SH_0 wave (Figure S8 f,g).

Figure S9 explicates the experimental SH_0 wave reception performance of co-fired quasi-d_{36} 7-layers specimen. The SH_0 signals are emitted by d_{24} specimens and successfully directly received in broadband frequencies by an oscilloscope. In fact, the apparent quasi-d_{36} response also inherently derives from inverse piezoelectric effect of d_{31} mode similar to the situation in Section S4. CWT results in time domain can produce wave information by calculating corresponding group velocity. Assuming the interval between emitted and received impulse signals is t_{CWT}, the group velocity v_g can be calculated as

$$v_g = D/t_{CWT} \quad \text{(Equation S11)}$$

where D is the distance between actuator and sensor (here is 360 mm). To verify the successful generation wave packet of co-fired quasi-d_{36} specimen, t_{CWT} is observed as 119.8 μs (Figure 5d), and v_g is calculated to be 3005 m/s. This value is in agreement with theoretical v_g value of 3099 m/s for SH_0 wave in the aluminum plate.

However, a multilayer structure usually weakens voltage response derived from thinner effective piezoceramic layers and bigger effective capacitance, so to directly (or after simple voltage amplification) detect its voltage signals for sensing is not a good choice. For a co-fired quasi-d_{36} multilayer structure ($t_{single\ layer}$ is the thickness of each single layer) under equivalent strain x_6, theoretical voltage output V_{eff} presents as

$$V_{eff} = \frac{e_{36} x_6 t_{single\ layer}}{\varepsilon} \quad \text{(Equation S12)}$$

where e_{36} and ε denotes the equivalent piezoelectric stress constant of quasi-d_{36} structure and dielectric parameter of component piezoceramics, respectively. It can be seen that multilayer d_{36} sensors will produce

smaller sensing voltage under the same deformation due to the thinner thickness ($t_{single\ layer}$) of each layer (The phenomenon can also be explained by amplified inner impendence).

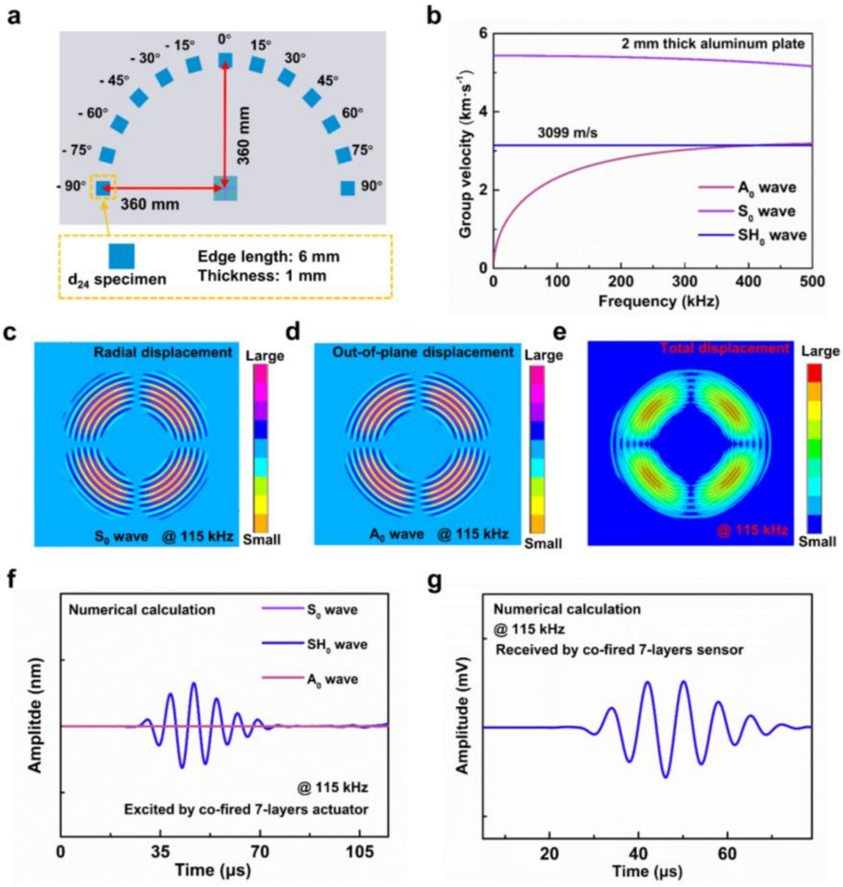

Figure S8. Dimension details of measurement system and FEM simulation for SH_0 wave generation and reception performance of co-fired specimen. **a**) Layout and geometrical dimensions of testing system. **b**) Group velocity curves of the Lamb wave (S_0 and A_0 waves) and SH_0 waves in the 2 mm thick aluminum plate, where the group velocity of SH_0 is perfectly nondispersive. **c-e)** S_0 wave (c), A_0 wave (d) and total displacement (e) simulated by finite element method. **f**, **g**) Numerical results of SH_0 wave excitation (f) and reception (g) performance of co-fired d_{36} specimen.

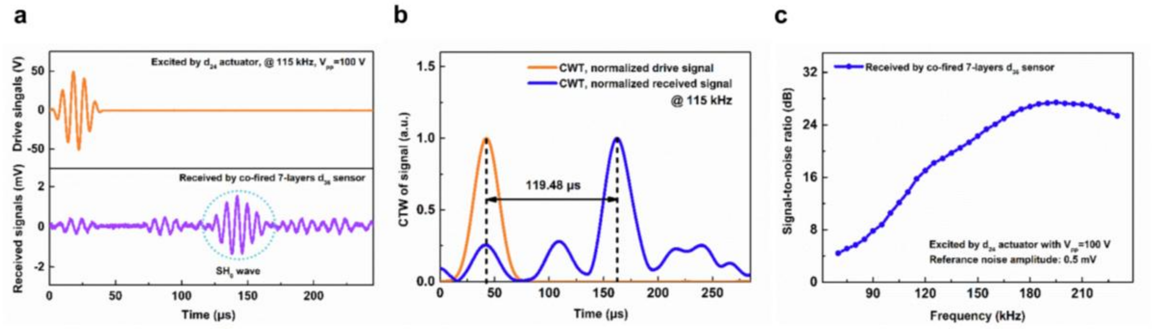

Figure S9. Experimental SH_0 wave reception performance of co-fired specimen as a sensor. **a**, **b**) Wave signals excited by d_{24} structure and received along the principal direction by 7-layers d_{36} structure (a), and corresponding time domain signals by continuous wavelet transform (CWT) at 115 kHz (b). **c**) Received signals as a function of working frequencies.

Section S6. Theoretical analysis of enhanced sensing capabilities

In Section S5, the voltage response of the co-fired d_{36} 7-layers sensor is directly detected by an oscilloscope without any preamplification. Usually, output signals of piezoelectric sensors are pretty weak, so they should be pre-amplified before further processing and analyses. There exist two kinds of mechanism: through voltage amplifier where output voltages are proportional to input voltages (namely, the voltage outputs of sensors); through charge amplifier where output voltages are proportional to input charge (namely, the charge outputs of sensors). During practical applications, the variable input impedance relevant to length and types of cables will lead to serious deviation from initial calibration, and substantially ruin the reliability of practical piezoelectric sensors in voltage amplification method. On the contrary, the voltage response of charge amplification way won't be affected by these factors and also become the mainstream technology to process the original signals of piezoelectric sensors.

Although a multilayer piezoelectric sensor usually weakens voltage output derived from thinner effective piezoceramic layers and amplified effective capacitance (as the analysis in Section S5), its capability of generating piezoelectric charge should be greatly enhanced. Theoretically, the firstly-created co-fired shear mode sensor will contribute to better sensing signals because of its amplified charge output from multilayer structure.

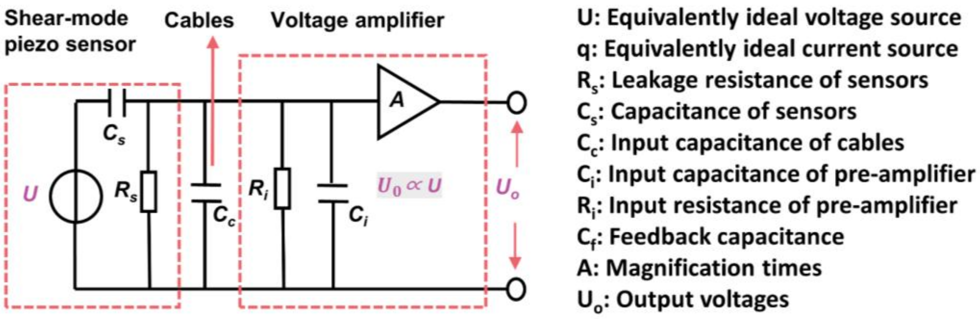

Figure S10. Voltage detection way for piezoelectric sensors. With the aid of voltage amplifier, the output voltage is proportional to original voltage output of sensor. All physical quantities here and for charge-detection way in Figure 5g are detailly explained.
